# Goats work for food in a contrafreeloading task

**DOI:** 10.1038/s41598-020-78931-w

**Published:** 2020-12-21

**Authors:** K. Rosenberger, M. Simmler, C. Nawroth, J. Langbein, N. Keil

**Affiliations:** 1 Swiss Federal Veterinary Office, Centre for Proper Housing of Ruminants and Pigs, Agroscope, 8356 Ettenhausen, Switzerland; 2 Graduate School for Cellular and Biomedical Sciences, University of Bern, 3012 Bern, Switzerland; 3 Digital Production Group, Agroscope, 8356 Ettenhausen, Switzerland; 4 Leibniz-Institute for Farm Animal Biology, Institute of Behavioural Physiology, 18196 Dummerstorf, Germany

**Keywords:** Animal behaviour, Statistical methods

## Abstract

Contrafreeloading (CFL) is the phenomenon when animals work for a resource although an identical resource is available for free. Possible explanations for CFL are that animals seek context for species-specific behaviours or to control their environments. We investigated whether goats show CFL and whether breeding for productivity traits has altered its occurrence. In a manipulation task, we compared two selection lines: 27 Nigerian dwarf goats, not bred for productivity traits, and 30 dairy goats, bred for high milk yield. Over 10 trials, each goat could perform one of three behaviours: not participating in the trial, feeding for free from an open door, or opening a sliding door for a feed of similar value. The results were analysed using an Item Response Tree (IRTree) generalized linear mixed model (GLMM). The fitted probabilities to participate were > 0.87 over all trials in both selection lines. For dwarf goats, the probability of choosing the closed door, and thereby demonstrating CFL, increased from 0.30 in Trial 1 to 0.53 in Trial 10. For dairy goats, this probability was constant at approximately 0.43. Unlike dwarf goats, dairy goats were faster to approach the closed compared to the open door. Overall, our results suggest that both selection lines were similarly interested in CFL.

## Introduction

Contrafreeloading (CFL) describes the phenomenon when animals, given the choice, work for a resource even though an identical resource is simultaneously available for free^1–3^. This phenomenon contradicts optimal foraging theory (e.g.^4^) which suggests that an animal will maximize the net energy gain by choosing the food source providing most energy for the lowest cost^5^. Although CFL is documented in captive wildlife and domestic pigs, cattle, goats, and chicken^6–9^, it has not been reported in animals living in the wild^10^. One prominent theory to explain the occurrence of CFL is the Information Primacy Model^2^. It assumes that CFL is driven by the urge to gather information about optimal food sources in a natural environment where food shortages can occur. As a result, if food deprivation increases, optimal foraging strategies will increase and the preference to gather information decreases. The need to explore the environment might, therefore, be an important adaptive mechanism^10^ and possibly explain why CFL occurs in animals in captivity^2^ where food is abundant.

Another theory to explain CFL is White’s Competence Theory^11^ which is not mutually exclusive from the Information Primacy Model. White’s theory postulates the need of animals to control and modify their surroundings and assumes that the successful performance of a task reinforces itself^1,6,12^ by increasing the perceived control over the environment^2,13,14^. Several studies found that mastering a task can induce positive emotions in farm animals^15–19^ that often live in barren environments with little stimulation or possibilities to control their surroundings. For example, in a study by De Jonge et al.^6^, pigs preferred searching for food rewards in straw rather than receiving the identical rewards freely available from a trough. The authors concluded that the display of CFL could be best explained by the rewarding effect of the anticipation of food while foraging. Therefore, if the need to control aspects of the environment and/or the need to perform species-specific behaviours are the motivators behind CFL, providing tasks to satisfy CFL motivation within a farm setting might enhance animal welfare. To effectively enrich housing conditions, all individuals of a species should frequently take part in the CFL task.

The type of training used, and the characteristics of the task offered to measure CFL may affect the proportion of individuals participating in the particular CFL experiment. Meagher et al.^20^ assessed the motivation to learn in cattle. They trained 30 heifers to perform an operant response (nose touch) to access a compartment providing a discrimination task. Although they used positive reinforcement (clicker training), the authors had to omit 10 animals mainly due to poor engagement in the task during the training phase. Participation proportions in the discrimination learning task varied between individuals and ranged from 0–100% of the offered sessions. Also, in studies where all of the individuals participated, huge individual differences in the extent of CFL display were found (i.e.^6,21–23^). For example, in a study with starlings, the percentage of choosing to work for food ranged from 0–100% across individuals^21^, whereas, in a study on pigs, the relative fraction of ‘earned’ food from total food consumed ranged from 0.4–12%^24^. Similar variations in CFL levels were reported in goats by Langbein et al.^25^. In this study, 12 dwarf goats were trained to operate an automated learning device that posed a discrimination task to receive drinking water. They found that 10 out of 12 goats chose to direct, on average, one-third of their total daily button presses towards the device instead of pressing the normal drinker where no prior discrimination task had to be solved. Some goats gained more than 80% of their daily water at the learning device, while other goats gained very little water at the device.

It has been postulated that tasks that resemble the species’ foraging behaviours or need low effort to receive the reward will promote CFL^2,6^. Goats are considered an intermediate ruminant type in the line of browsing—grazing species^26^. They feed on a mixture of shrubs, trees, and grasses, often switching seasonally^27^, and browse for the most nutritive fractions in their food^28,29^. In contrast to sheep and cattle, which are mainly grazers, the time spent browsing can make up to 73–93% of their feeding time, depending on season^27^. A suitable CFL task for goats might resemble this natural browsing behaviour, allowing for oral manipulation of the test apparatus. Additionally, a low-effort and easily executable manipulation task is expected to increase the number of individuals frequently choosing to work for the reward^2^.

Domestication in general, and the selection for high productivity in particular, were found to have altered not only stress reactivity^30–32^, but also foraging^7,8,33,34^ and exploration behaviour^31^ in farm animals. Compared to less productive breeds, pigs selected for high feed efficiency showed less behavioural reactivity towards fear-eliciting stimuli and displayed increased latencies when approaching a novel object and an unfamiliar human^31^. Such alterations in behaviour may reflect in the motivation to show CFL. Schütz and Jensen^33^ compared White leghorn chicken, selected for high egg productivity, to red jungle fowl, the ancestor of the domestic chicken, and to Swedish bantam, a domestic breed not strongly selected for production traits. They found that White leghorn chicken obtained a lower proportion of their food through CFL than both junglefowl and bantam chicken. Selection for high egg productivity in White leghorn chicken might thus have either directly reduced their motivation for CFL or indirectly decreased traits such as curiosity and risk-taking that are likely to affect the preferences of animals to perform CFL. Whether selection for high milk yield had a similar effect on the motivation to show CFL is yet unknown. To address this question, goats may represent a suitable species as selection lines differ strongly in milk production performance. Goats specifically selected for the dairy industry, such as Saanen goats, can produce up to 2–3 kg of milk per day^35^. On the other hand, common pet goats such as Nigerian Dwarf goats were not selected for productivity traits and their milk yield is much lower (0.3 kg per day)^36^.

We examined whether domestic goats show CFL and repeatedly do so over several trials in a low-effort manipulation task that resembles their natural foraging behaviours. We provided goats with the choice between receiving a desired food item at an open door or opening a sliding door to access an identical food item. As some dwarf goats readily worked for more than 80% of their daily water intake in a previous CFL study^25^, we expected that goats in our experiment would also show CFL to a certain proportion, i.e., push the closed sliding door open to receive the food reward instead of choosing the free reward. If goats are more motivated to work for a reward instead of receiving it for free, we would expect the approach time towards the closed door to be shorter than towards the open door^20^. Following up on the findings by Schütz and Jensen^33^, who showed lower motivation for CFL in the high-productivity chicken line, we assessed whether selection for high milk yield had a similar effect on CFL in goats. We compared dairy goats with a pedigree for high milk production to Nigerian dwarf goats that have not been selected for productivity traits. To increase genetic variability in our sample, we used individuals of two dairy breeds and of their crossbred (Saanen, Chamois Colored, Saanen × Colored). Using an Item Response Tree (IRTree) generalized linear mixed model (GLMM)^37^, the experiment was statistically modelled as a sequence of binary decisions between mutually exclusive behaviours (participate or not, choose open or closed door, approach fast or slow). Additionally, a linear mixed model, with approach time as a continuous response, was used to compare approach times between the open and the closed door.

## Material, animals and methods

### Location, animals, and housing conditions

The study was carried out in August 2018 at the Agroscope Research Station in Ettenhausen, Switzerland. In total, we housed 60 domestic goats from two different selection lines: 30 Nigerian dwarf goats and 30 dairy goats. The Nigerian dwarf goat is commonly kept as pet and zoo animal in Europe and not selected for productivity traits. We used dwarf goats bred at the Leibnitz Institute for Farm Animal Biology in Dummerstorf, Germany. The only selection aim in this population was to avoid inbreeding, and the potential milk yield of dwarf goats does likely not exceed 0.3 kg per day^36^. All dwarf goats were born in January/February 2017. As it was common practice at the institute in Dummerstorf, dwarf goat kids stayed with their dams for 6 weeks before they were separated. They were moved to Ettenhausen in June 2017. To investigate the effect of selection for high productivity on CFL, we compared dwarf goats to dairy goats with a pedigree for high milk production. To increase genetic variability in our sample^38^, we used two of the most common high-producing dairy breeds in Switzerland and their crossbred, namely Saanen (n = 15), Chamois coloured (n = 12), and Saanen × Chamois (n = 3, see Supplementary Data S2). These breeds have a potential milk yield of up to 3 kg per day^35^. All dairy goats were born on Swiss farms in February to April 2017. In accordance with common practice in the dairy goat industry, the dairy goat kids from Swiss farms were separated from their dam shortly after birth and artificially raised. They were moved to Ettenhausen in June/July 2017.

In Ettenhausen, dwarf and dairy goats were initially housed in one group pen each. At the age of 7–8 months, all goats were moved to pens of 10 goats each: three groups of dairy goats and three groups of dwarf goats. The total area of each dwarf goat pen was 14 m^2^ (approximately 3.6 m × 3.9 m), consisting of a deep-bedded straw area of 11 m^2^ (approximately 2.8 m × 3.9 m) and a 0.5 m elevated feeding place (3.0 m^2^). The total area of each dairy goat pen was 17.5 m^2^ (approximately 3.9 m × 4.5 m), consisting of a deep-bedded straw area of 13.2 m^2^ (approximately 4.5 m × 2.9 m) and a 0.6 m elevated feeding place (4.3 m^2^). Hay was provided ad libitum behind a feeding fence and replenished twice a day at approximately 8 am and 4 pm. Each pen had one drinker and a mineral supply. Additional structures in the straw-bedded area included a wooden bench (dairy: 2.4 m long, 0.6 m high, 0.6 m wide; dwarf: 2.3 m long, 0.5 m high, 0.5 m wide) along the wall of the pen and a round wooden table (dairy: 0.8 m high, Ø 1.1 m; dwarf: 0.6 m high, Ø 1 m) in the centre of the pen. The goats were between 15 and 18 months old at the start of the study (mean ± SD, dairy goats: 529 ± 18.7 days, dwarf goats: 578 ± 4.7 days).

All procedures involving animal handling and treatment were approved by the Swiss Cantonal Veterinary Office Thurgau (Approval No. TG04/17—29343) and were performed in accordance with all relevant Swiss legislative and regulatory requirements and the ASAB/ABS guidelines for the use of animals in research^39^.

### Test apparatus and test arena

The goats were individually tested in a test arena (4.5 m × 2 m). The test apparatus was installed as part of a wall on the shorter side of the test arena and consisted of two identical wooden sliding doors. A metal grid fence (1.35 m high × 1.27 m long) was installed between the two sliding doors to prevent goats from switching sides and feeding from both openings right after each other (Supplementary Fig S1). A human experimenter (E1) was positioned behind the wall of the test apparatus. Whenever a goat opened the closed door (= CFL) or stuck its muzzle through the open door, it instantly received a piece of uncooked pasta in a plastic dish from E1 as a reward (see Supplementary Video).

### Habituation phase

The goats were familiar with the arena and with opening a sliding door from a previous experiment on social learning, and additional training in case it was necessary. In the previous experiment the apparatus consisted of one sliding door only. All goats had gone through a 3-day habituation phase to get used to feed from the open door: on day 1, goats had been habituated in pairs for 3 min and 10 pieces of pasta per pair of goats. On day 2 and 3, goats had been habituated individually over 3 min and 10 pieces of pasta per goat. In the subsequent test sessions, goats first observed a human demonstrator opening the closed sliding door and were then given the opportunity to open the door themselves. All goats had received a total of 10 sessions over 10 consecutive days. At the end of the experiment, all but four dwarf goats had learned to open the closed sliding door.

In the current experiment, we presented two identical sliding doors simultaneously. We provided no additional habituation to this novel setup as the previous experiment finished only two days prior to the start of the current experiment. The four dwarf goats that had not learnt to open the sliding door in the previous experiment received additional individual training on the day before the current experiment by leaving a slowly shrinking gap to facilitate door opening until the sliding door was fully closed. However, only one of the four goats successfully learned to open the door and, thus, was included in the study. The other three were excluded from the experiment. Consequently, we included 57 goats in our study, 30 dairy and 27 dwarf goats.

### Test procedure

**Figure 1:**
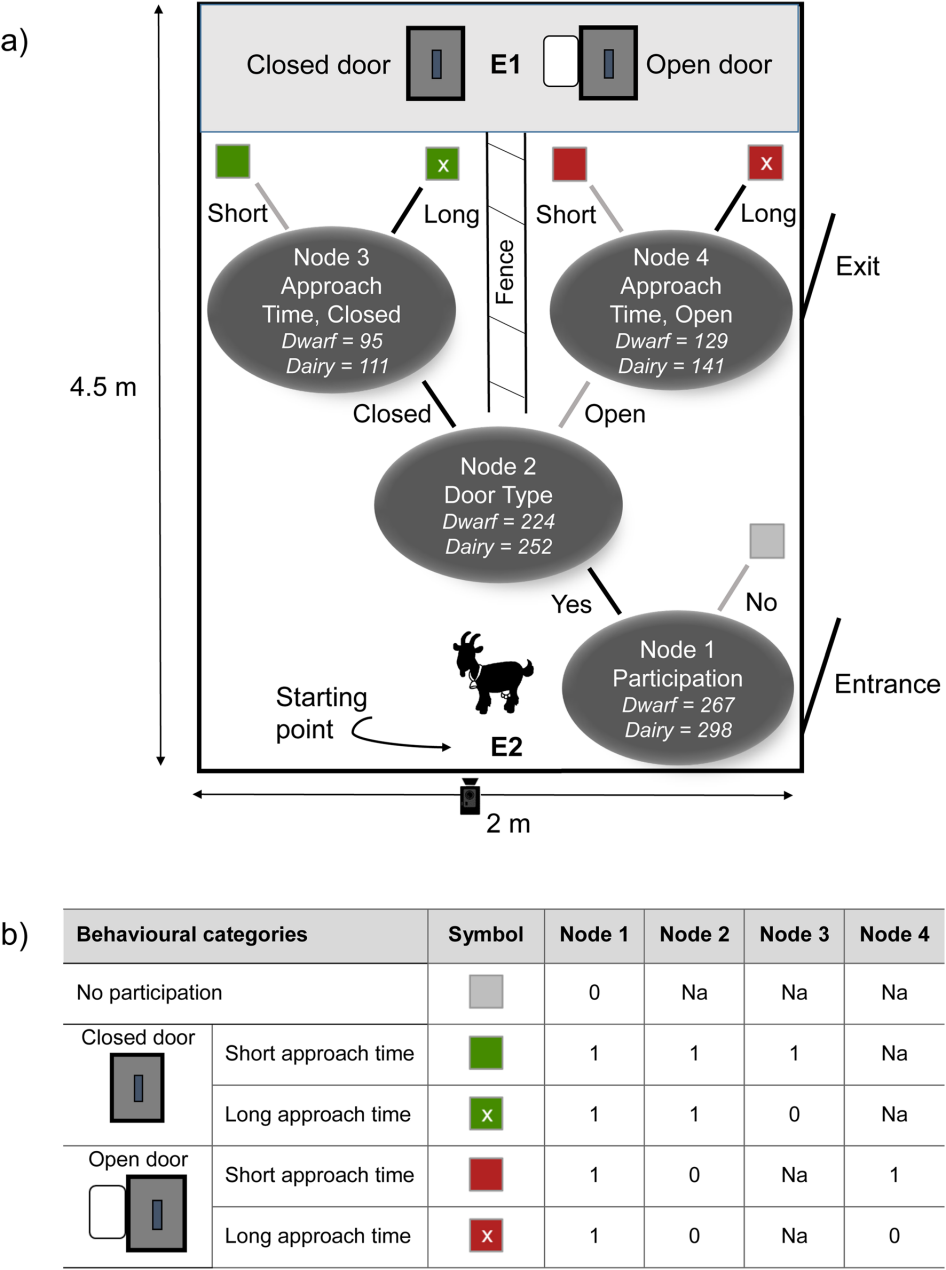
(**a**) Schematic drawing of the test arena with left door closed and right door open including the binary response tree with four nodes representing the sequential choices leading to one of five behavioural categories (= five square symbols). A number of observations with non-missing information at respective nodes are given in the tree. Positions of the experimenters are indicated with E1 and E2, and the position of the video camera is marked with a camera symbol. (**b**) The five behavioural categories with their symbols corresponding to the tree in (**a**) as well as the encoding of the node for the IRTree model.

Each goat received 10 consecutive trials on a single day with each trial lasting 30 s. In total, goats were tested within four days. In each trial, one of the two sliding doors was kept open, allowing free access to a food reward. The other door was presented closed, requiring manipulation to slide the door to the right or left to access the food, i.e. necessitating work for the reward (Fig. 1a, Supplementary Fig. S1). The closed door was administered on the left and the right sides of the fence in a pseudorandom order, but each goat was constrained to a total of five trials with the closed door on the left and five trials with the closed door on the right side.

Goats were individually led into the test arena by a second experimenter (E2) and released near the centre of the room approximately one meter away from the start of the fence (= starting point, Fig. 1a). In each trial, the goat could choose to walk to the closed or the open door. After each trial, the individual was led back to the starting point by E2, and the next trial started. All trials were videotaped with a camcorder (Sony HDR-CX240E) mounted on top of the wall of the arena above the starting point (Fig. 1a). Due to technical failures, five trials were not videotaped and therefore excluded from the analysis. The videos were analysed with the Observer XT software (Version 12, Noldus Information Technology, The Netherlands). We recorded whether the goat participated or not, and, if it participated, which door type it chose (closed = CFL or open = no CFL), as well as the time it took to approach the door (= nose in a distance of less than approximately 5 cm to the door) from the start of the fence (approach time, in sec). Participation was defined as walking towards one of the doors, opening the door, if closed, and feeding through the door from the plastic dish. If a goat did not participate within 30 s after it was released, the trial was recorded as ‘no-participation’, and the goat was led back to the starting point to begin the next trial. Opening the closed door without feeding from the dish was never observed. To assess the reliability of the approach times determined from videos, we compared them to times recorded during the experiment with a stopwatch (Pearson correlation coefficient, r_p_ = 0.85). Participation and the choice of door type were unambiguous.

### Data analysis and statistics

All statistical analyses were performed in R v4.0.2^40^. We employed an Item Response Tree (IRTree) model of the GLMM family to analyse the multivariate behavioural response in our experiment. Such IRTree models allow representing a multivariate behavioural response as a tree of sequential binary responses, enable incorporating hierarchical sampling, and can account for correlated responses as well as repeated testing of the same individuals^37^. They are well suited for analysing categorical data in behavioural studies, as shown for example for escalating courtship behaviours, antipredator behaviours, and social interactions^41,42^.

For encoding the data as a binary tree and specification of the model, we followed recommendations by López-Sepulcre et al.^41^ (see Supplementary Data S1). Figure 1a shows the binary response tree of our IRTree model. The nodes correspond to the sequential choices (responses) leading to one of five behavioural categories (= five symbols). Node 1 encodes the participation in a given trial (‘yes’, 1; ‘no’, 0). Node 2 encodes the choice of either walking to feed at the open (0) or the closed (1) door. Finally, Nodes 3 and 4 encode the time taken to walk from the start of the fence to the closed or the open door, respectively. This approach time was short (0) or long (1) corresponding to below or above the median over all observations of the respective selection line (dwarf = 2.66 s, dairy = 1.81 s; Supplementary Fig. S2). Goats that chose not to participate in a trial made only one choice while participating goats made three sequential choices, as Node 3 and 4 are mutually exclusive (see Fig. 1a,b). The IRTree model was estimated as GLMM with a binary response and logit link using the *glmer* function from the R package lme4^43^. The model formula in *lme4* syntax was as follows:

$${\text{Value }}\sim \, 0 \, + {\text{ Node:SelectionLine }} + {\text{ Node:SelectionLine:I}}\;({\text{Trial }} - { 1}) \, + \, ({\text{1 | Obs}}) \, + \, (0 \, + {\text{ Node | Pen/Individual/ClosedSide}})$$

The nodes were qualitatively different from each other, and we suspected the selection lines to differ in their behaviour as well as to adapt their responses with repeated trial. As fixed effects, we therefore, included for each node an individual intercept for the two selection lines (0 + Node:SelectionLine) and an individual slope for the trial number for the two selection lines [Node:SelectionLine:I(Trial − 1)]. The trial number was included as Trial − 1 to render the intercept to correspond to Trial 1 instead of the non-meaningful Trial 0. As a random effect, we included a random intercept for observation (1|Obs) to ensure that the sequential binary responses corresponding to a single observation shared the same variance and were not treated as independent observations. Furthermore, we specified for each node a random intercept for the location of the closed door, nested within individual, itself nested within pen (0 + Node|Pen/Individual/ClosedSide). This was done to account for potential side bias, repeated testing of the same individual, and potential effects of pen affiliation. Despite the randomization of the location of open and closed door, a side bias was evident; the choice for the closed door was apparently more likely when the closed door was on the left versus the right side (Wald test in repeated measure logistic regression, p = 0.03).

To investigate the difference in behaviour between the selection lines, we tested selection line contrasts for the fixed effects using the *glht* function from the R package multcomp^44^. Both the p-values for fixed effect estimates (*glmer* function) and for the contrasts (*glht* function) were obtained using Wald tests. Fitted probabilities and bootstrap confidence bands that were only conditioned on the fixed effects were obtained using the *predict.MerMod* function (parameter re.form =  ~ 0; lme4 package) in conjunction with the *bootMe*r function (lme4) for parametric bootstrapping (10,000 bootstraps).

To compare approach times towards the open versus the closed door, we also analysed the approach time as a continuous response in a linear mixed model using the *lmer* function (lme4 package) and the following model formula:

$${\text{log2}}\;({\text{ApproachTime}}) \, \sim \, 0 \, + {\text{ SelectionLine }} + {\text{ SelectionLine:DoorType }} + {\text{ SelectionLine:DoorType:I}}\;\left( {{\text{Trial}} - {1}} \right) \, + \, \left( {\text{1 | Pen/Individual/ClosedSide}} \right)$$

The approach time was right-skewed and, therefore, log2-transformed to approximate normal distribution (Supplementary Fig. S2). Individually for the two selection lines, we included an intercept (0 + SelectionLine), an effect of the choice of door type (SelectionLine:DoorType), and a slope for trial separately for the closed and open doors [SelectionLine:DoorType:I(Trial − 1)]. Besides these fixed effects, a random intercept for the location of the closed door, nested within individual, itself nested within pen (1|Pen/Individual/ClosedDoor) was included to account for potential side bias, repeated testing of the same individual, and potential effect of pen affiliation. P-values for the fixed effect estimates were obtained using Z-tests through the *glht* function (multcomp package). The R code used to perform the analyses described in this section is provided as Supplementary Material (Supplementary Code S1).

## Results

**Figure 2:**
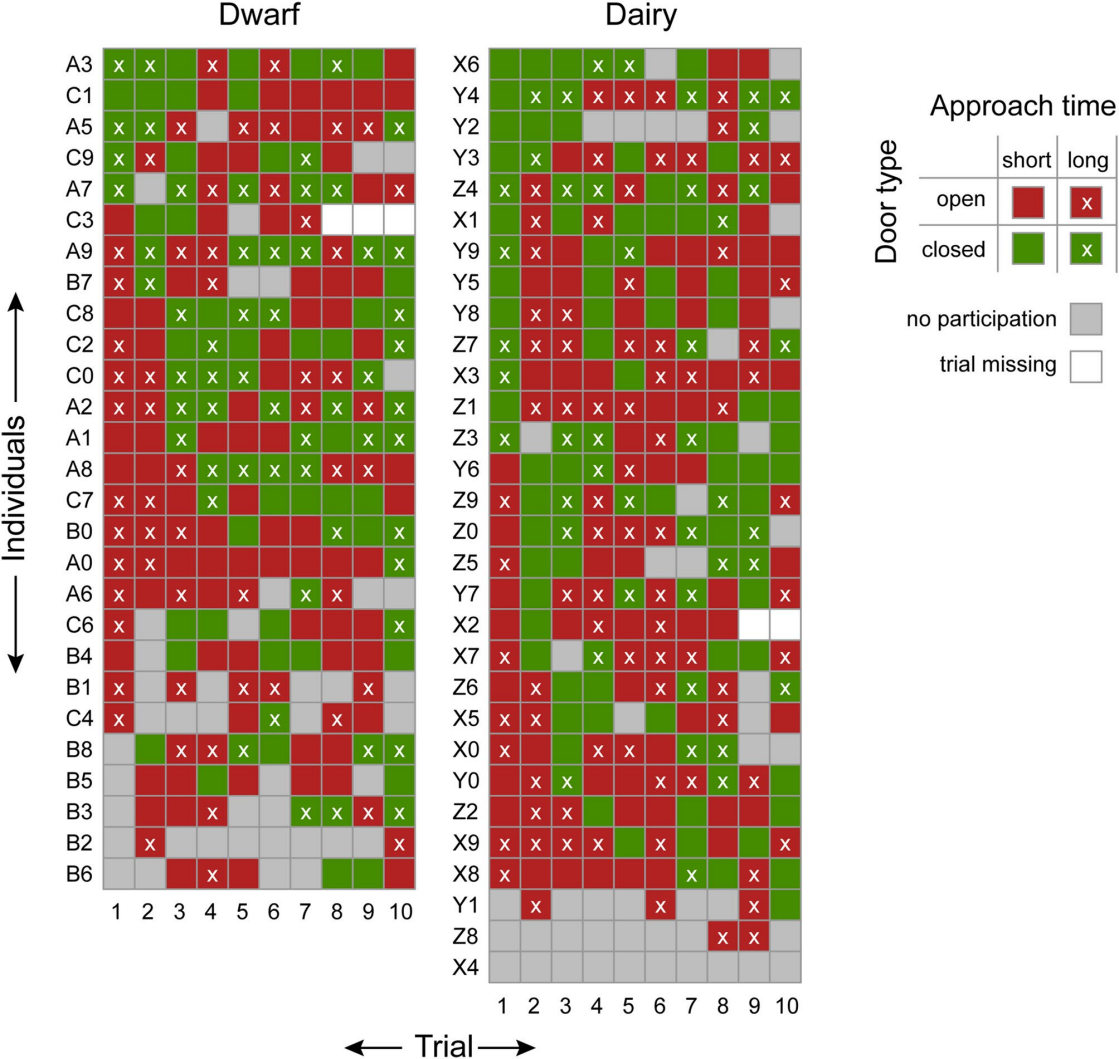
Individual distribution of choices regarding door type and approach time from trials of dwarf and dairy goats. Names of individuals are composed of a letter for the pen affiliation (dwarf: A, B, C; dairy: X, Y, Z) and a number for the individual within each pen (0–9).

We found that all but four goats chose to participate in at least half of the 10 trials of the experimental task. Three individuals participated in less than 5 of 10 trials (B2, Y1, Z8) and only one individual chose in all trials to not participate (X4, Fig. 2). In total, 53 of 57 goats chose to feed from the closed door in at least 1 of 10 trials, but inter-individual variation was substantial with values ranging from 1 to 7 of the 10 trials (Fig. 2).

**Figure 3:**
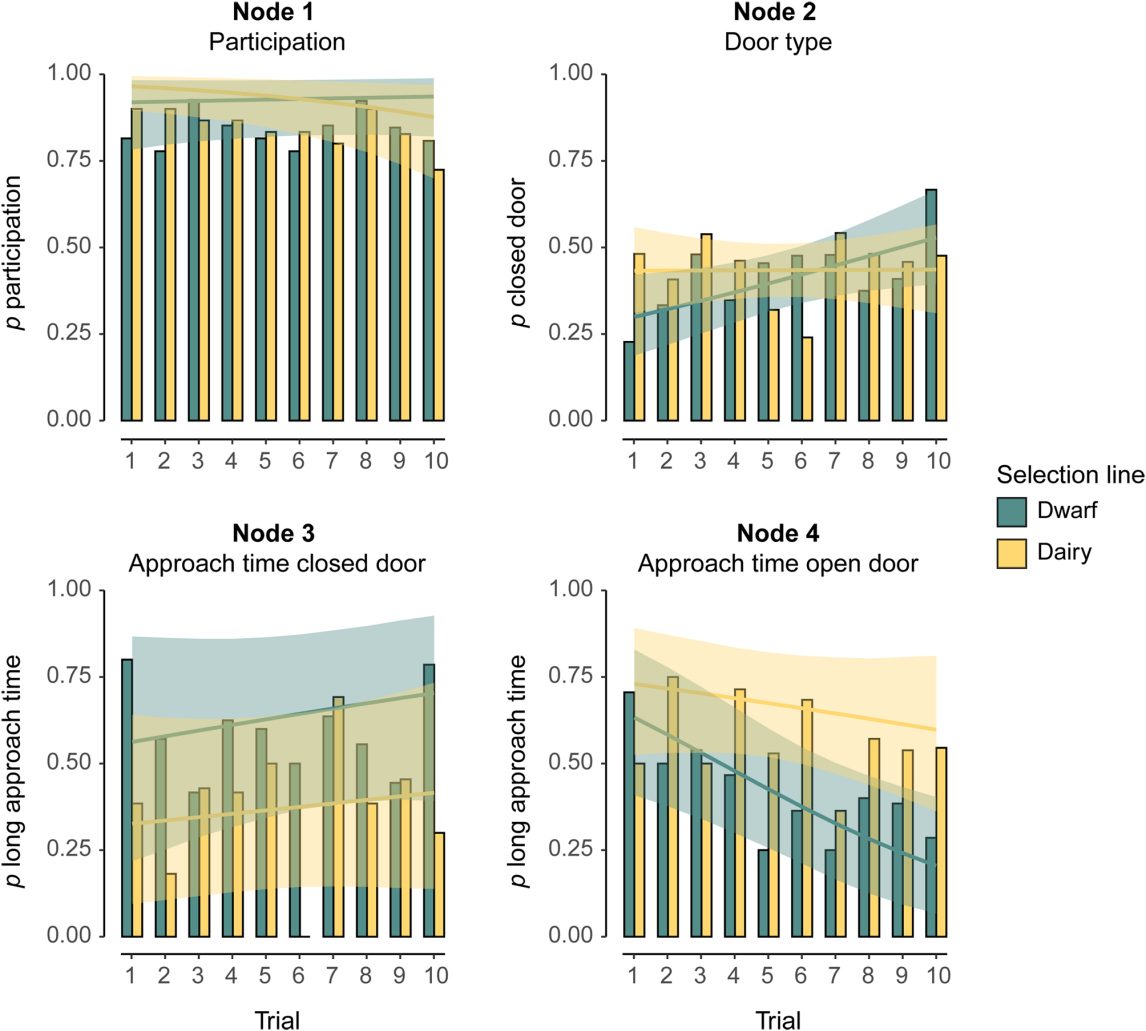
Fitted probabilities (lines) of the IRTree GLMM at the four nodes and observed proportions (bars). The shaded areas represent 95% confidence bands for the fitted values considering the fixed effect uncertainty.

The results of the IRTree GLMM are presented in Table 1 (and Supplementary Table S1 and S2). Fitted probabilities of behavioural choices represented by the nodes as well as observed proportions are shown in Fig. 3. The fitted probability to participate in the task was > 0.87 over all trials for both selection lines (Fig. 3—Node 1). In dairy goats, but not in dwarf goats, the probability of participating decreased as the trial numbers increased—from 0.97 in Trial 1 to 0.88 in Trial 10 (p = 0.04, Table 1, Fig. 3—Node 1).

**Table 1:** IRTree GLMM of behavioural responses. Intercepts correspond to Trial 1 (see “Methods”). Results with p-value ≤ 0.05 are given in bold. An extended version of this table, including standard error and Z-values, is provided in the Supplementary Material (Table S1). Additionally, Supplementary Table S2 lists the random effect variance components and correlations.

Fixed effects	Node 1: Participation *p* participation				Node 2: Door type *p* closed door				Node 3: Approach time closed *p* long approach time				Node 4: Approach time open *p* long approach time			
	Dwarf		Dairy		Dwarf		Dairy		Dwarf		Dairy		Dwarf		Dairy	
	est	p	est	p	est	p	est	p	est	p	est	p	est	p	est	p
Intercept	**2.43**	<** 0.001**	**3.32**	<** 0.001**	**− 0.85**	**0.003**	**− **0.27	0.29	0.25	0.75	**− **0.72	0.27	0.55	0.25	**0.99**	**0.04**
(Trial − 1)	0.03	0.69	**− 0.15**	**0.04**	**0.11**	**0.03**	0.002	0.97	0.07	0.48	0.04	0.57	**− 0.21**	**0.01**	**− **0.07	0.34

Contrast in…	Dwarf–dairy				Dwarf–dairy				Dwarf-dairy				Dwarf-dairy			
	est		p		est		p		est		p		est		p	
Intercept	0.90		0.32		0.58		0.13		− 0.98		0.33		0.45		0.50	
(Trial − 1)	− 0.18		0.08		− 0.11		0.12		− 0.03		0.84		0.15		0.17

In dwarf goats, the probability of choosing the closed door was 0.30 in Trial 1, but it increased with increasing trial number (p = 0.03) and reached a probability of 0.53 in Trial 10 (Fig. 3—Node 2). In dairy goats, the probability of choosing the closed door was approximately constant at around 0.43 throughout all trials (Fig. 3—Node 2).

The probability for a long approach time (= above the median over all trials of the respective selection line) towards the closed door ranged from 0.56 to 0.70 in dwarf goats and from 0.33 to 0.42 in dairy goats (Fig. 3—Node 3). As the uncertainty in these probabilities was high (Fig. 3—Node 3, Table 1), a difference between selection lines is statistically not supported (p = 0.33 for intercept contrast in Trial 1).

In dwarf goats, the probability for a long approach time towards the open door decreased with increasing trial number (p = 0.01)—from 0.63 in Trial 1 to 0.20 in Trial 10 (Fig. 3—Node 4). In dairy goats, the probability for a long approach time towards the open door was 0.73 in Trial 1 and 0.60 in Trial 10 (Fig. 3—Node 4), with a high uncertainty in these probabilities throughout the various trials (p = 0.34).

**Figure 4:**
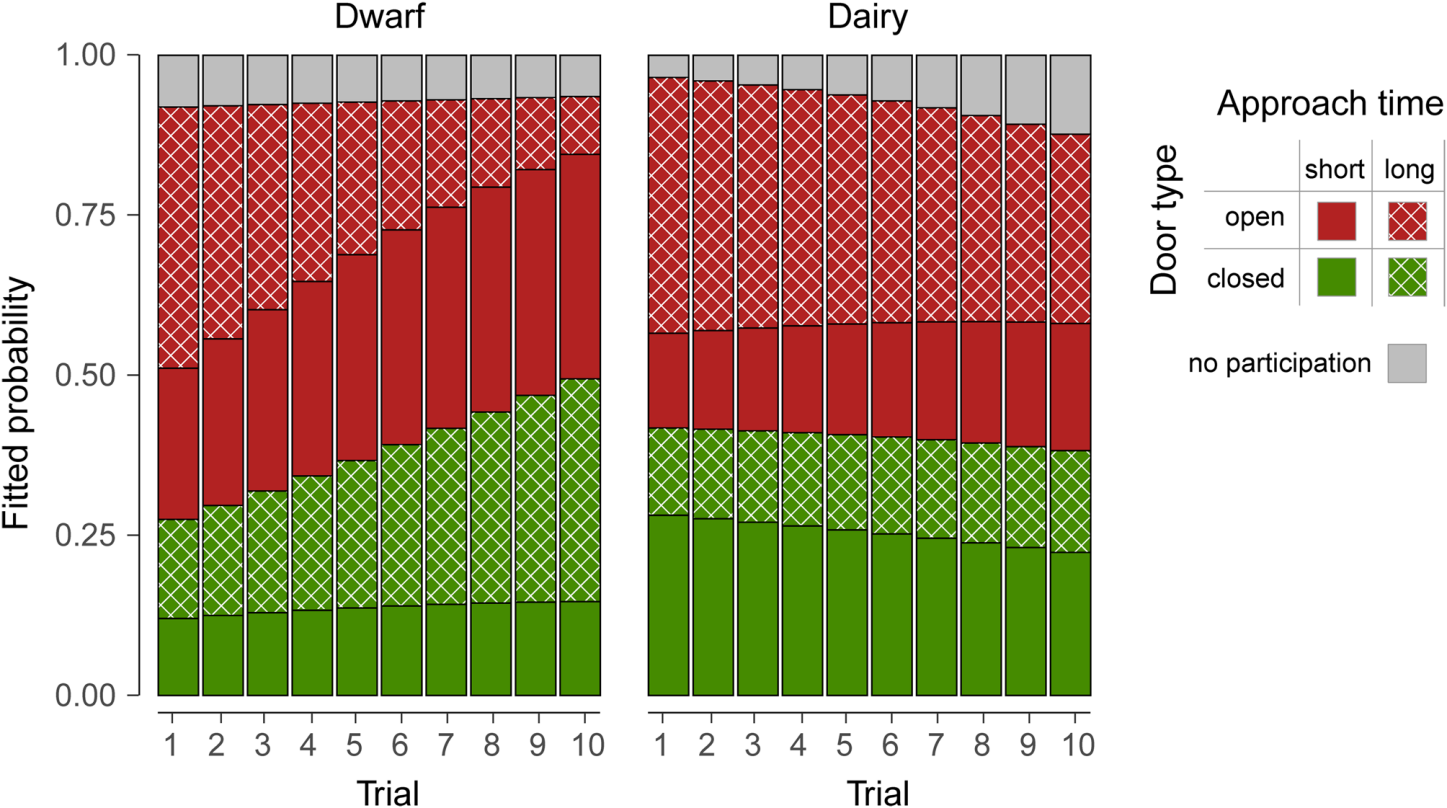
Fitted probabilities of the IRTree GLMM for the five behavioural categories. These probabilities were calculated by multiplying the probabilities of the corresponding sequential choices (Fig. 1a,b). For example, the probability for the behavioural category ‘open door, long approach time’ (Figs. 1a,b, 3) was calculated as the probability to participate (Node 1), times the probability to choose the open door (Node 2), times the probability to show a long approach time (Node 4).

Figure 4 shows the probabilities of the five behavioural categories, representing the possible outcomes of the sequential choices. In dwarf goats, the probability for ‘open door, long approach time’ decreased with increasing trial number in favour of the probabilities for ‘open door, short approach time’ and for ‘closed door, long approach time’. In dairy goats, the probability for ‘open door, long approach time’ decreased in favour of the probability for ‘no participation’.

**Figure 5:**
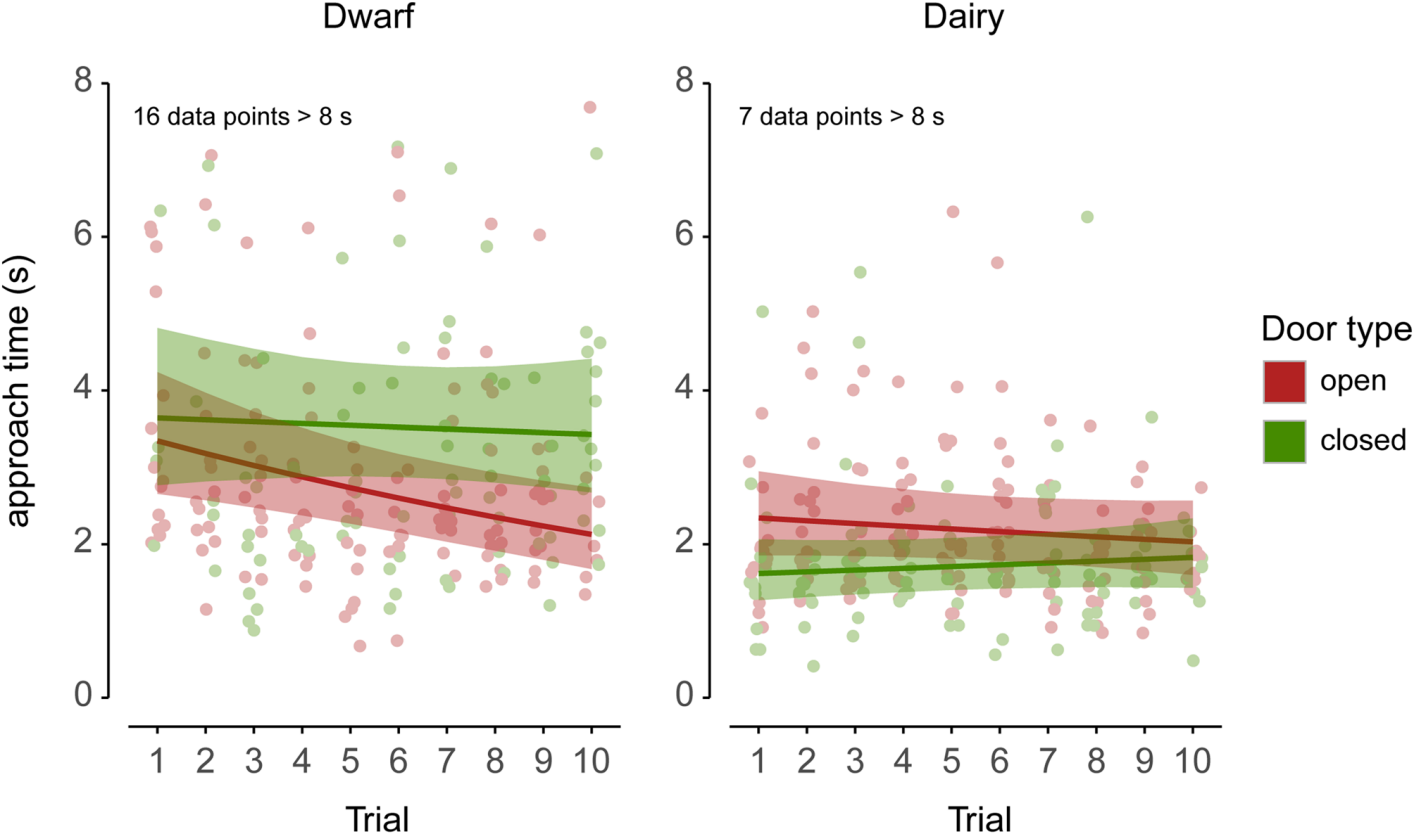
Fitted approach times of the linear mixed model (back-transformed from log2 to linear scale). The shaded areas represent 95% confidence bands for the fitted values considering the fixed effect uncertainty. Dots represent observed approach times. For each trial number, the dots were horizontally jittered for visual clarity. Data points > 8 s are not shown (numbers given in panels).

The results of the linear mixed model, with approach time as continuous response, are presented in Table 2 (and Supplementary Table S3). Fitted approach times are shown in Fig. 5. Unlike the IRTree model above, this model allows a direct comparison of approach times towards the open versus the closed door. The fitted approach time for the dwarf goats towards the closed door was approximately constant at around 3.5 s (Fig. 5). Towards the open door, the fitted approach time was 3.3 s in Trial 1, decreased over time (p = 0.002), and was 2.1 s in Trial 10 (Fig. 5).

**Table 2:** Estimates of variance components and fixed effects of the LMM with continuous approach time as response. Intercepts correspond to Trial 1 (see “Methods”). Results with p-value ≤ 0.05 are given in bold. Supplementary Table S3 lists the random effect variance components.

Fixed effects	Dwarf				Dairy			
	est	s.e.	z	p	est	s.e.	z	p
Intercept	**1.73**	**0.17**	**10.05**	<** 0.001**	**1.21**	**0.17**	**7.05**	<** 0.001**
Door type closed	0.12	0.20	0.60	0.55	− **0.54**	**0.18**	− **3.04**	**0.002**
(Trial − 1): door open	− **0.07**	**0.02**	− **3.14**	**0.002**	− 0.02	0.02	− 1.01	0.31
(Trial − 1): door closed	− 0.01	0.03	− 0.34	0.73	0.02	0.02	0.81	0.42

For dairy goats, the fitted approach time towards the closed and the open door was approximately constant over the trials and ranged from 1.6 (Trial 1) to 1.8 s (Trial 10) and 2.3 (Trial 1) to 2.0 (Trial 10), respectively. Thus, dairy goats approached the closed door faster than the open door (p = 0.002).

## Discussion

In line with our hypothesis, goats chose to participate in the experiment and were willing to work for a reward in the presence of an identical, free reward, and thus chose to perform CFL. Contrary to our expectations, the selection line was not related to the overall probability of working for food but rather to changes in goats’ responses with increasing trial numbers. Our results suggest that both selection lines were motivated to work for food, while this motivation was suppressed in early trials in dwarf goats, presumably due to higher stress reactivity.

The high proportion of goats that frequently chose to participate in the experiment suggests that we used a suitable experimental setting with a highly desired reward and enough previous habituation to the test arena and the sliding door in order to make participation rewarding for goats. To avoid spatial learning, we randomized the location of the open and the closed door in all trials. However, we found indications that the closed door was more likely to be chosen when located on the left side. As the goats’ pen mates where located on the right side, this bias seems not socially induced. In our statistical models we considered the potential side bias as a random effect nested in individual, thus allowing an individual side bias for each goat.

In accordance with our expectations, all but four goats exhibited CFL in at least 1 of 10 trials. Overall, the probabilities to choose the closed door were not different between the dwarf and dairy goats. This is in contrast to what we expected and to previous studies on different selection lines of chicken^8,33,34^ and cattle^7^ which found that animals selected for high productivity were choosing more energy conserving strategies and thus were less likely to show CFL than animals not selected for production traits. In our experiment, the probability in dwarf goats to choose the closed door increased with increasing trial number, while it remained approximately constant for dairy goats (Fig. 3—Node 2). As all goats were familiar with receiving food out of both doors, open and closed, the initial reluctance to choose CFL in dwarf goats is unlikely to be explained by neophobia towards one of the doors^45^. Although all goats had been habituated to the single sliding door in a previous experiment, no additional habituation to the novel setting with two sliding doors was performed in the current experiment. This new setting may have induced more stress in dwarf goats than in dairy goats and resulted in dwarf goats initially choosing the option that appears to be less risky (i.e. the open door). Increasing habituation and positive reinforcement from opening the door might then have increased motivation to choose CFL in subsequent trials. This is in line with the notion that stress reactivity has been reduced in animals selected for high productivity^33,46^, which would suggest reduced stress reactivity in dairy goats compared to dwarf goats.

However, not only a genetic disposition but also differences in rearing may have caused different stress responses between the selection lines in our experiment. Whereas the dairy goats were artificially raised without their mothers, the dwarf goats stayed with their dams for 6 weeks. Previous studies^47,48^ found indications for higher fearfulness of dam-reared goats in goat-human encounters as compared to human-reared goats—dam-reared goats exhibited greater behavioural responsiveness in novel situations, as well as longer latencies to approach an unfamiliar human.

Regarding the effect of door type on approach times, we hypothesised that, if goats are motivated to work for a reward instead of receiving it for free, they would approach the closed door faster than the open door^20^. Our results only support this hypothesis in dairy goats, which approached the closed door faster. In contrast to dairy goats and not in line with our hypothesis, dwarf goats showed similar approach times towards both doors in the first few trials and tended to approach the open door faster in later trials (Fig. 5). An explanation for these observations may again be differences in stress reactivity. Maybe dwarf goats would have required more time to adapt to the test situation to react similarly to dairy goats regarding their approach time towards the closed and open door. Recent research on farm animal personality highlights the need to consider the animals’ individual stress levels for their habituation to experimental tasks^49^, regardless of whether it is genetically based or developed during the ontogenesis.

Over all trials, the probability of choosing CFL was slightly below 50%, raising the question whether our results could partially be explained by goats randomly choosing the open or closed doors. However, we found the probability of choosing CFL in dwarf goats to increase over trials and the approach time to be affected by door type. This indicates that goats deliberately chose the door type rather than choosing at random. An explanation for the occurrence of CFL over several trials might be attributed to intrinsic rewarding properties of the performance of the task itself ^1,6,12^. Positive emotions as a result of mastering a task and being in control of the situation have been reported in cattle, pigs, and goats^15–19^. This is also in line with White’s Competence theory^11^, which postulates that animals are motivated to manipulate and control their environment to attain competence. Hence, it is possible that goats were choosing CFL due to positive feedback from executing the manipulation task.

It has been suggested that animals need appropriate cognitive challenges to express control over their environment^15–19^ and that animals value effort^50^ such that the incorporation of such challenges into a farm setting can have welfare benefits for the animals^15,17,51^. For a successful implementation of CFL tasks in the husbandry system, it would be necessary to evaluate if the motivation to display CFL is stable in various conditions and also persists over a longer period of time. The high inter- and intra-individual variations in the extent of CFL, which are in accordance with other studies on CFL in dwarf goats^25^ and in other species^21,23,52^, require further research regarding the motivational background to show CFL in animals.

## Conclusion

Overall, high CFL proportions in both selection lines, increasing interest in approaching the closed door in dwarf goats, and shorter approach times towards the closed door compared to the open door in dairy goats indicate that both dairy and dwarf goats were motivated to work for a resource in the presence of the same resource for free. The two selection lines of goats differed in the changes of the probabilities to choose CFL with increasing trial number and regarding the comparison of approach times towards the open versus towards to closed door. These results might reflect differences in stress reactivity towards the CFL task, potentially related to selection for productivity or differences during ontogeny. Our findings suggest that goats seem to be motivated to solve a CFL task, stressing the need for the provision of cognitive challenges to improve the welfare of farm animals.

## Supplementary Information

Supplementary Information 1.

Supplementary Information 2.

Supplementary Information 3.

Supplementary Information 4.

Supplementary Video 1.

## Data Availability

All data generated or analysed in this study are included in this article (and its Supplementary Information files).
