# Supplementary material for: Goats work for food in a contrafreeloading task
Source: Sci Rep. 2020 Dec 21;10:22336. doi: 10.1038/s41598-020-78931-w (PMC7752918; doi:10.1038/s41598-020-78931-w)
Supplement: Supplementary file 1 — Supplementary Information 1. [file 41598_2020_78931_MOESM1_ESM.pdf]

Supplementary Information

for

Title:

Goats work for food in a contrafreeloading task.

Authors:

Rosenberger K., Simmler M., Nawroth C., Langbein J., and Keil N.

Table S1: IRTree GLMM of behavioural responses. Intercepts correspond to Trial 1 (see Methods). This table is an extended version of Table 1 in the paper. Results with p-value ≤ 0.05 are given in bold.

	**Node 1: Participation** *p* participation								**Node 2: Door type** *p* closed door							
	Dwarf				Dairy				Dwarf				Dairy			
	est.	s.e.	z	p	est.	s.e.	z	p	est.	s.e.	z	p	est.	s.e.	z	p
Intercept	**2.43**	**0.64**	**3.77**	**<0.001**	**3.32**	**0.69**	**4.82**	**<0.001**	**-0.85**	**0.29**	**-2.92**	**0.003**	-0.27	0.26	-1.05	0.29
(Trial -1)	0.03	0.07	0.39	0.69	**-0.15**	**0.07**	**-2.07**	**0.04**	**0.11**	**0.05**	**2.14**	**0.03**	0.00	0.05	0.03	0.97
	**Dwarf - Dairy**								**Dwarf – Dairy**							
Contrast in…	est.		s.e.		z		p		est.		s.e.		Z		p	
Intercept	0.90		0.90		0.99		0.32		0.58		0.38		1.51		0.13	
(Trial – 1)	-0.18		0.10		-1.77		0.08		-0.11		0.07		-1.55		0.12	
	**Node 3: Approach time closed door** *p* long approach time								**Node 4: Approach time open door** *p* long approach time							
	Dwarf				Dairy				Dwarf				Dairy			
	est.	s.e.	z	p	est.	s.e.	z	p	est.	s.e.	z	p	est.	s.e.	z	P
Intercept	0.25	0.78	0.32	0.75	-0.72	0.65	-1.11	0.27	0.55	0.48	1.15	0.25	**0.99**	**0.48**	**2.08**	**0.04**
(Trial -1)	0.07	0.10	0.71	0.48	0.04	0.08	0.57	0.57	**-0.21**	**0.08**	**-2.65**	**0.008**	-0.07	0.07	-0.95	0.34
	**Dwarf - Dairy**								**Dwarf - Dairy**							
Contrast in…	est.		s.e.		z		p		est.		s.e.		z		p	
Intercept	-0.98		1.01		-0.97		0.33		0.45		0.66		0.68		0.50	
(Trial – 1)	-0.03		0.12		-0.21		0.84		0.15		0.10		1.38		0.17	

Table S2: Random effect variance components and correlations of the IRTree GLMM.

**Random effects**						
**Groups**	**Name**	**Variance**	**Std.Dev.**	**Corr**		
Obs	(Intercept)	0.00	0.00			
ClosedSide:(Individual:Pen)	NodeN1	0.25	0.50			
	NodeN2	0.22	0.47	0.34		
	NodeN3	0.09	0.30	0.33	-0.77	
	NodeN4	0.15	0.39	1.00	0.39	0.28
Individual:Pen	NodeN1	2.90	1.70			
	NodeN2	0.00	0.0.03	1.00		
	NodeN3	1.00	1.00	-0.33	-0.33	
	NodeN4	1.14	1.07	-0.77	-0.77	0.86
Pen	Node 1	0.36	0.60			
	Node 2	0.01	0.10	1.00		
	Node 3	0.64	0.80	1.00	1.00	
	Node 4	0.10	0.31	1.00	1.00	1.00

Table S3: Random effect variance components of the LMM with continuous approach time as response.

**Random effects**			
**Groups**	**Name**	**Variance**	**Std.Dev.**
ClosedSide:(Individual:Pen)	(Intercept)	0.02	0.14
Individual:Pen	(Intercept)	0.13	0.36
Pen	(Intercept)	0.03	0.18
Residual		0.53	0.73

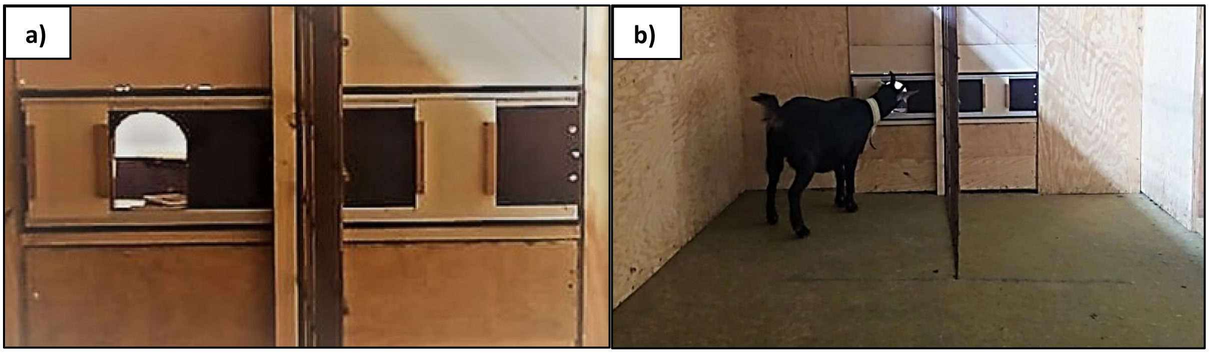

Figure S1: a) Test apparatus with left sliding door open (= free reward) and right sliding door closed (= work). b) Test setup with dwarf goat feeding from the open door.

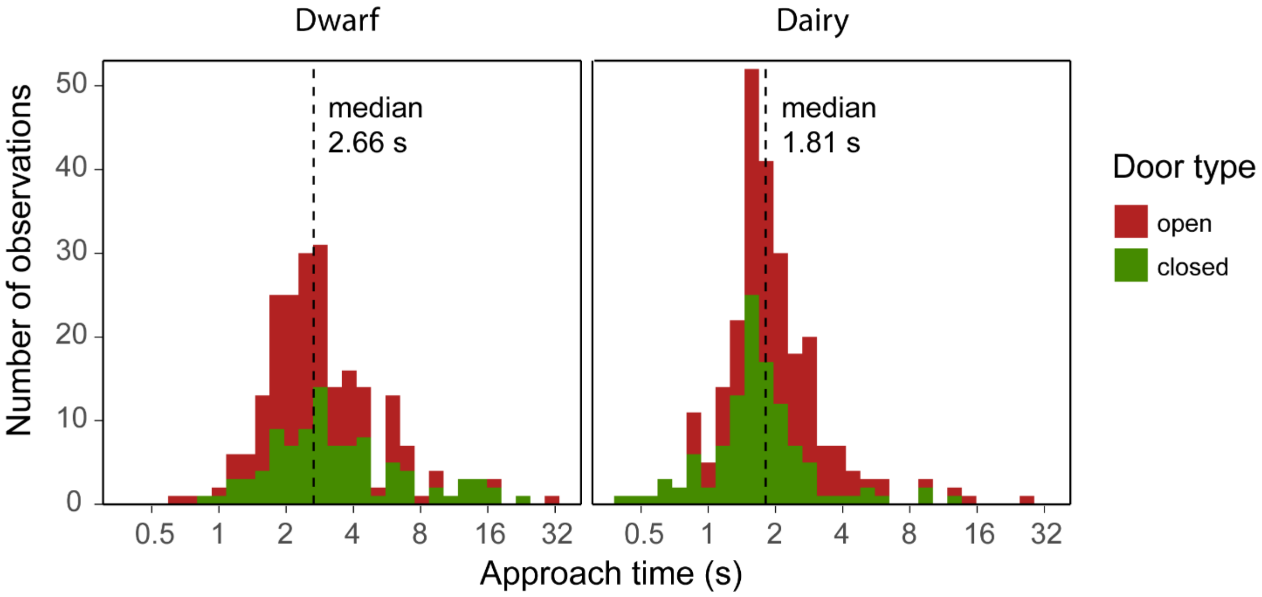

Figure S2: Distribution of approach times of dwarf and dairy goats towards the open and closed doors.
